# Supplementary material for: Simultaneous Detection of Exosomal microRNAs Isolated from Cancer Cells Using Surface Acoustic Wave Sensor Array with High Sensitivity and Reproducibility
Source: Micromachines (Basel). 2024 Feb 7;15(2):249. doi: 10.3390/mi15020249 (PMC10892992; doi:10.3390/mi15020249)
Supplement: Supplementary file 1 [file micromachines-15-00249-s001.zip › micromachines-2792632-supplementary.pdf]

Simultaneous Detection of Exosomal microRNAs Isolated from Cancer Cells Using Surface Acoustic Wave Sensor Array with High Sensitivity and Reproducibility

Su Bin Han and Soo Suk Lee*

Department of Pharmaceutical Engineering, Soonchunhyang University, 22 Soonchunhyang-ro, Shinchang-myeon, Asan-si, Chungcheongnam-do, 31538, Republic of Korea; 1gkstkfkd@naver.com (S. B. H)
* Correspondence: sslee0810@sch.ac.kr (S.S.L); Tel.: +82-41-530-1394

1) Optimization of the concentration of the immobilized capture probe

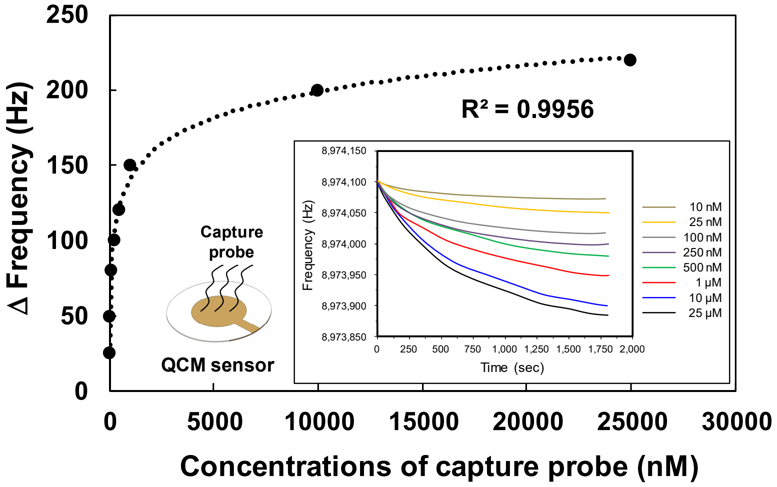

Figure S1. Concentration optimization of the immobilized capture probe (complementary sequence to the miR-21, 5'-H_2N-$(CH_2)_6$-TCA ACA TCA GTC TGA TAA GCT ACC CGG GCC CG-3') tested using a 9 MHz quartz crystal microbalance (QCM) resonator. When the probe concentration reaches 25 μM, saturation begins to occur.

2) Gel electrophoresis images of sandwich hybridization assay

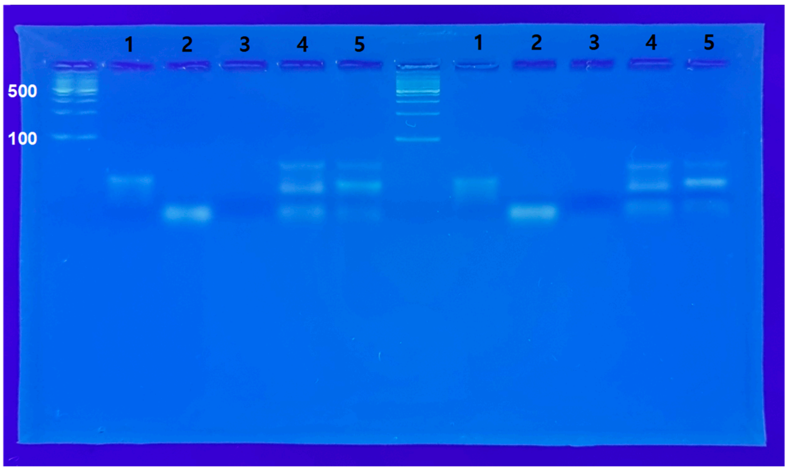

Figure S2. Gel electrophoresis images of sandwich hybridization assay. lane 1, capture probe (complementary sequence to the miR-106b, 31 bps); lane 2, miR-106b (21 bps); lane 3, detecting probe (10 bps); lane 4, parcial hybridization (1 + 2); lane 5, sandwich hybridization (1 + 2 +3).

3) Comparison of changes in resonance frequency depending on the labeling method

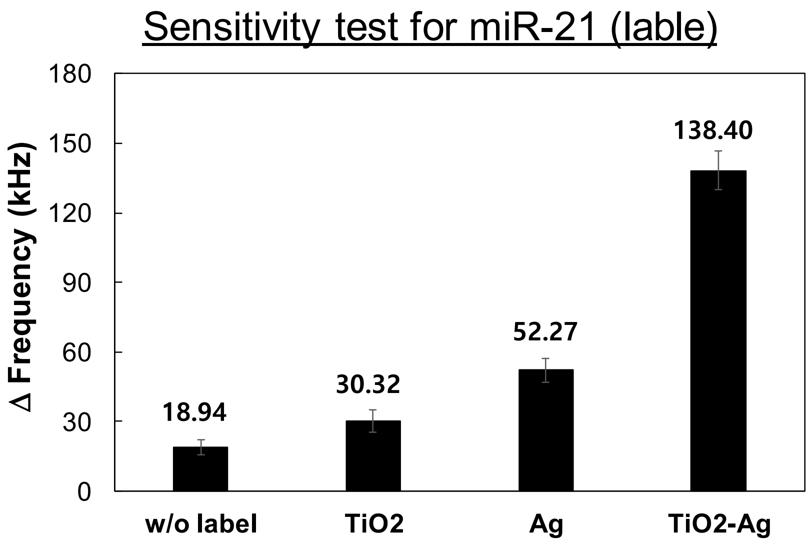

Figure S3. Comparison of changes in resonance frequency of SAW biosensors for detecting miR-21 depending on the labeling method to the detecting probe. The labeling of TiO_2 nanoparticles and photocatalytic silver staining show the largest signal change compared to other methods. The concentration of miR-21 was 10 nM.

4) The result of the sensor without target RNA (A blank test)

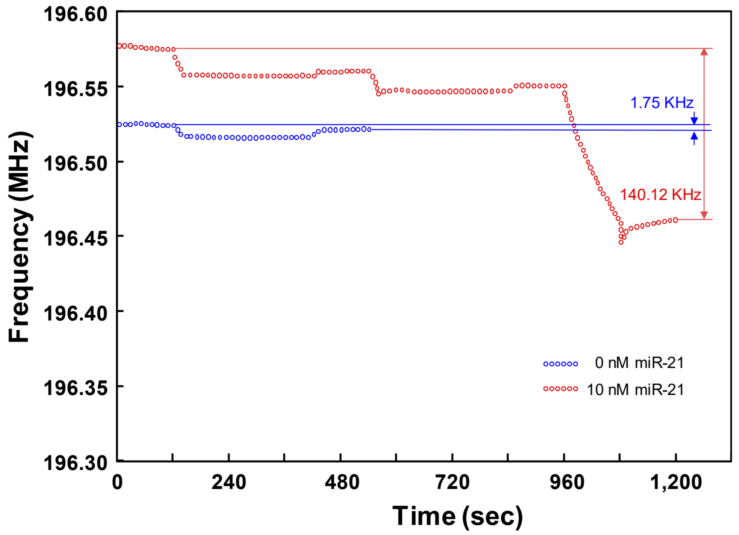

Figure S4. SAW sensor response due to sandwich hybridization and subsequent TiO_2-mediated silver staining reaction. A decrease in frequency indicates an increase in the effective mass of the sensor chip. Blank (blue dotted line) and 10 nM concentration of the synthetic miR-21 (red dotted line) was used in this experiment.

5) Selectivity of the SAW sensor array

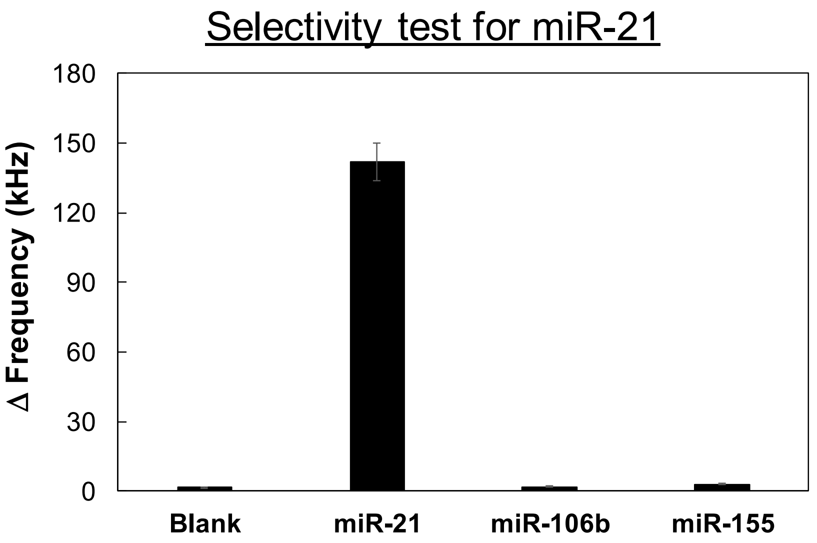

Figure S5. Selectivity of the SAW biosensor toward miR-21 in comparison with two other miRNA samples. miR-21 shows a more than 50 times larger signal compared to the other non-complementary miRNAs (miR-106b and miR-155) and blank sample. The concentrations of four miRNAs were 10 nM.
